# Effect of Different Sodium Hypochlorite Concentrations on Post Endodontic Pain: A Systematic Review and Meta‐Analysis of Randomized Controlled Trials

**DOI:** 10.1002/cre2.70320

**Published:** 2026-02-24

**Authors:** Kavalipurapu Venkata Teja, Kaligotla Apoorva Vasundhara, Romeo Patini, Salvatore Scolavino, Gianrico Spagnuolo, Flavia Iaculli, Mariangela Cernera

**Affiliations:** ^1^ Department of Conservative Dentistry and Endodontics Mamata Institute of Dental Sciences Hyderabad India; ^2^ Saveetha Dental College and Hospitals, Saveetha Institute of Medical and Technical Sciences Saveetha University Chennai India; ^3^ Department of Head, Neck and Sense Organs Catholic University of Sacred Heart Rome Italy; ^4^ Department of Neurosciences, Reproductive and Odontostomatological Sciences University of Naples Federico II Naples Italy; ^5^ Department of Oral and Maxillofacial Sciences Sapienza University of Rome Rome Italy

**Keywords:** endodontic flare‐up, irrigation, post‐endodontic pain, root canal treatment, sodium hypochlorite

## Abstract

**Objective:**

The aim of the present systematic review and meta‐analysis was to evaluate the effect of different concentrations of sodium hypochlorite (NaOCl) on post‐operative pain following root canal treatment.

**Material and Methods:**

The current study was conducted according to PRISMA guidelines and registered on PROSPERO (CRD42023451576). The research question was formulated according to PICO strategy and aimed at determining if different concentrations of NaOCl would have influenced post‐operative pain in adults following endodontic treatments. A comprehensive literature search was performed manually and through multiple electronic databases, using predefined keywords and MeSH terms. Randomized controlled trials (RCTs) comparing different NaOCl concentrations (< 5% vs. ≥ 5%) and reporting post‐operative pain in adults were included. The risk of bias was assessed using the Cochrane RoB 2 tool. Meta‐analyses were conducted for pain occurrence at 24, 48, and 72 h post‐treatment.

**Results:**

Seven RCTs met the inclusion criteria and were included in the systematic review; moreover, four studies underwent quantitative evaluation (meta‐analysis). The results showed no statistically significant difference in post‐operative pain between low (< 5%) and high (≥ 5%) NaOCl concentrations at any time point. However, a low concentration of NaOCl seemed to be associated with less pain, significantly in 2 papers and not significantly in 4 articles out 7. Only one study reported the opposite trend.

**Conclusion:**

Within the limitations of the present study, no significant differences in post‐endodontic pain were found between high and low NaOCl concentrations. However, concentrations < 5% might be associated with less pain incidence. Further well‐designed clinical trials with standardized methodologies are needed to provide more conclusive evidence.

## 1 Introduction

Endodontic treatment should not only focus on microbial elimination from the root canal system but also on mitigating the pain caused by various preoperative and postoperative factors (Smith et al. 2017). Pain management should take into account a variety of pre, intra and postoperative factors (Arias et al. 2013). Several variables such as type of irrigant, irrigation activation, extrusion of irrigants (Liapis et al. 2021; Karatas et al. 2021), flare‐up (Bassam et al. 2021), instrumentation techniques (Western and Dicksit 2017), debris extrusion (Adiguzel et al. 2019), occlusal reduction (Nguyen‐Nhon et al. 2020) could influence pain during and after the endodontic procedure. In addition, some mechanical factors might provoke post‐operative pain including enlargement of apical foramen (Silva et al. 2013), failure to maintain apical patency (Yaylali et al. 2018), over instrumentation of the root canal, movement kinematics (Adiguzel et al. 2019) and root canal sealers (Ferreira et al. 2020).

Evidence has proven that primary endodontic infections are predominated by gram positive microorganisms (Chávez de Paz 2004; Narayanan and Vaishnavi 2010). Prostaglandins (PGs)—a major component of the cell wall—contribute to trigger and up regulate the inflammatory process (Grga et al. 2013). Indeed, PGs cause vasodilation, increase vascular permeability, decrease the threshold of nociceptors that on turn evoke post‐operative pain. Moreover, it's been reported that the presence of PGE2, PGF2α, and PGA2 has a significant role in generating endodontic pain (Siqueirajr et al. 2002). Lipoteichoicacid (LTA), a component of gram‐positive bacterial cell wall, has shown to influence inflammatory mediators (Park et al. 2013). *Enterococcus faecalis*, a gram‐positive bacteria, possess polyglycerol phosphate‐type LTA (EfLTA) that plays an important role in biofilm formation and bacterial adhesion (Hong et al. 2016). In addition, LTA induces proinflammatory mediators and instigate the release of proinflammatory cytokines such as Tumor Necrosis Factor alpha (TNF‐a), Interleukin‐1 (IL‐1), Interleukin‐ 8 (IL‐8), Interleukin‐12 (IL‐12) (Henderson 1996; Parolia 2015). These proinflammatory cytokines cause further release of substance P that might increase endodontic pain (Jain et al. 2013). Furthermore, the extrusion of dentinal debris during root canal treatment, in combination with microbial agents and chemical disinfectants, may induce an acute inflammatory reaction in the periapical tissues, thereby causing post‐endodontic pain (Teja et al. 2025). Sodium hypochlorite (NaOCl), the mostly used endodontic irrigant, presents several properties as antimicrobial effect, canals lubrication, smear layer formation prevention and pulp tissue dissolving, and it's been demonstrated to be effective in inactivating and eliminating LTA (Marending et al. 2007). However, its extrusion into the periradicular area may result in significant adverse tissue reactions and pain due to its caustic nature (Prasad et al. 2024). Although it's been demonstrated that higher NaOCl concentrations are associated with post‐operative pain occurrence (Prasad et al. 2024; Sabino‐Silva et al. 2023), its optimal safe concentration as well as the persistence of pain within the first 72 h should be further elucidated. Therefore, the aim of the present systematic review and meta‐analysis was to assess the influence of different concentrations of NaOCl on post‐operative pain following root canal treatment.

## 2 Material and Methods

The current systematic review was prepared in agreement with the PRISMA guidelines statement (Page et al. 2021) and the review protocol was listed on PROSPERO (CRD42023451576).

The review addressed a focused question by using the participant, intervention, comparison, outcomes, study design (PICOS) criteria as follows: “Does different concentrations of sodium hypochlorite influence post‐operative pain in adults following root canal treatment in randomized clinical trials?”

Population: Healthy subjects > 18 years old.

Intervention: Root canal treatment of dental elements with necrosis or irreversible pulpitis.

Comparison: Different concentrations of sodium hypochlorite used during root canal treatment.

Outcome: Post‐operative pain incidence.

Study: Randomized clinical trials.

### 2.1 Search Strategy

A comprehensive literature search was conducted through multiple electronic databases (PubMed, Scopus, Web of Science, EMBASE, Cochrane) up to December 2024 using specific keywords and Mesh terms as reported in Table 1. A manual search restricted to papers published between January 2000 and December 2024 was also conducted in these peer‐reviewed journals dealing with endodontic sciences: Journal of Endodontics, International Endodontic Journal, Australian Endodontic Journal, European Endodontic Journal, and Clinical Oral Investigation. Moreover, reference sections of the included articles were manually assessed to identify further papers for potential inclusion.

**Table 1:** Search strategy.

PubMed	((((((((((root canal irrigants) AND (sodium hypochlorite)) AND (different concentration)) OR (various concentration)) AND (post operative pain)) OR (post endodontic pain)) AND (randomized controlled trial))) AND (non randomized controlled trial)) AND (clinical study)
Web of Science	Root canal irrigants (All Fields) and sodium hypochlorite (All Fields) and different concentration (All Fields) or various concentration (All Fields) and post operative pain (All Fields) or post endodontic pain (All Fields) and randomized controlled trial (All Fields) and non‐randomized controlled trial (All Fields) and clinical study (All Fields)
Cochrane	Root canal irrigants in Title Abstract Keyword OR sodium hypochlorite in Title Abstract Keyword OR different concentration in Title Abstract Keyword AND post operative pain in Title Abstract Keyword AND clinical trial in Title Abstract Keyword
SCOPUS	(ALL (root AND canal AND irrigants) AND ALL (sodium AND hypochlorite) AND ALL (different AND concentration) AND ALL (post AND operative AND pain) OR ALL (post AND endodontic AND pain) AND ALL (randomized AND controlled AND trial) AND ALL (clinical AND study) AND ALL (non AND randomized AND controlled AND trial))
Embase	(((‘root canal irrigants’/exp OR ‘root canal irrigants’ OR ((‘root’/exp OR root) AND canal AND irrigants)) AND (‘hypochlorite sodium’/exp OR ‘hypochlorite sodium’) AND (‘different concentration’ OR (different AND (‘concentration’/exp OR concentration))) OR ‘various concentration’ OR (various AND (‘concentration’/exp OR concentration))) AND (‘post operative pain’/exp OR ‘post operative pain’ OR (post AND operative AND (‘pain’/exp OR pain))) OR ‘post endodontic pain’ OR (post AND endodontic AND (‘pain’/exp OR pain))) AND (‘randomized controlled trial’/exp OR ‘randomized controlled trial’ OR (randomized AND controlled AND (‘trial’/exp OR trial))) AND (‘non randomized controlled trial’ OR (non AND randomized AND controlled AND (‘trial’/exp OR trial))) AND (‘clinical study’/exp OR ‘clinical study’ OR ((‘clinical’/exp OR clinical) AND (‘study’/exp OR study)))

### 2.2 Eligibility Criteria

The papers were selected in agreement with the following criteria:

Inclusion criteria

– Papers published in Peer‐reviewed Journals;
– Full text articles published in English language;
– Randomized clinical trials evaluating the effect of different concentrations of sodium hypochlorite (NaOCl) on post‐operative pain in adult patients underwent non‐surgical root canal therapy;
– Randomized clinical trials conducted in accordance to Declaration of Helsinki guidelines developed by World Medical Association.

Exclusion criteria

– Animal studies, in vitro studies, review articles, case reports, and case series.
– Clinical and randomized controlled trials conducted on patients underwent retreatment.
– Gray literature.

### 2.3 Selection of Studies

Two independent calibrated reviewers (M.C. and F.I.) screened the retrieved papers and identified relevant studies after removing duplicates. Based on the eligibility criteria, studies were evaluated by title and abstract. Then, full texts of possibly relevant papers were retrieved and evaluated. Any disagreements between examiners were solved by a third investigator (G.S.). Agreement level within the two examiners was recorded using Cohen's kappa coefficient (k).

### 2.4 Data Extraction

Data extraction forms were independently filled by the two examiners (M.C. and F.I.), for further analysis. The following information was reported: authors, year, country, tooth group, patients' number, patients' age, sex, number of visits, temporary dressing, shaping instrument, coronal sealing, pre‐operative sealing, use of painkillers before and after root canal therapy, NaOCl percentages, additional irrigation protocol, obturation material, obturation technique, pain evaluation scale, follow‐up time and main findings. When pain levels were not reported as numbers, the data were extracted from bar graphs or provided images using a specific tool (https://automeris.io/).

### 2.5 Risk of Bias and Quality Assessment

Qualitative assessment was independently performed by two examiners (M.C. and F.I.) on the included papers by means of a revised Cochrane risk‐of‐bias tool for randomized trials (RoB 2) (Sterne et al. 2019) on Review Manager 5.2 software (RevMan, The Nordic Cochrane Centre, Copenhagen, Denmark). Five domains were addressed to report the risk of bias: randomization processes, deviations from intended interventions, missing outcome data, measurement of the outcome and selection of the reported result. Based on the above‐mentioned criteria, the studies were categorized as “low risk,” “some concerns,” or “high risk.” Overall low risk of bias for a study was given when all the domains were scored as “low risk.” “Some concerns” was given to study with at least one domain scored as “some concerns.” Finally, a study was considered to have an overall high risk of bias when at least one domain was scored as “high risk” or more than two domains were scored as “some concerns.” An additional analysis of the overall quality of evidence for each meta‐analysis was independently conducted using the Grading of Recommendations, Assessment, Development, and Evaluations (GRADE) system.

### 2.6 Data Synthesis

Data extraction was conducted independently and in duplicate by two examiners (M.C. and F.I.). Since the differences in reporting pain incidence among studies (i.e., pain scales applied differently), the pain occurrence was considered as present (summarizing together all levels of pain) or absent at every evaluated time points. Meta‐analyses were conducted with a fixed‐effect model only if the included studies were homogeneous in terms of study population demographics. Only in case of a not‐negligible heterogeneity across included articles, thus meaning clinical and methodological diversity across studies or the presence of studies with small sample size, a random‐effect model was used; moreover, a value of *I* ^2^ exceeding 50% was considered as predictive of non‐negligible heterogeneity. Meta‐analyses were conducted only for comparisons with at least four included studies.

### 2.7 Assessment of Heterogeneity and Statistical Analysis

The Review Manager (RevMan) software was used for assessing the heterogeneity across the included papers (The Nordic Cochrane Centre, The Cochrane Collaboration. Review Manager [RevMan] v. 5.2. Copenhagen, Denmark: The Nordic Cochrane Centre, The Cochrane Collaboration; 2013). The comparability of the observed differences across the results with chance alone was calculated by the authors using the *χ* ^2^ test and the *I* ^2^ test. Heterogeneity was considered significant if *p* value was < 0.1 (Review Manager 5.2 software—RevMan, The Nordic Cochrane Centre, Copenhagen, Denmark). Moreover, the *I* ^2^ test was considered as measure of heterogeneity across studies, as follows (Higgins et al. 2003):

– 0%–40%: negligible
– 30%–60%: moderate
– 50%–90%: substantial
– 75%–100%: considerable.

## 3 Results

### 3.1 Study Selection

**Figure 1:**
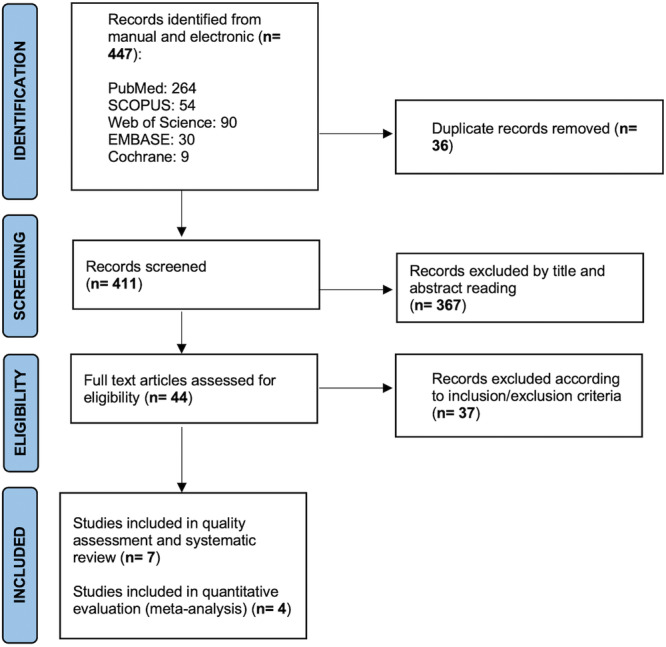
PRISMA flow‐chart.

The electronic and manual search resulted in 447 papers. After duplicates removal, a total of 411 records underwent evaluation by title and abstract. Then, full texts of the remaining 44 papers were retrieved and evaluated in agreement with the eligibility criteria. Finally, 7 papers were included in the current systematic review and processed for qualitative analysis (Farzaneh et al. 2018; Verma et al. 2019; Mostafa et al. 2020; Ulin et al. 2020; Demenech et al. 2021; Mokhtari et al. 2023; Vitali et al. 2024); in addition, 4 studies (Farzaneh et al. 2018; Verma et al. 2019; Demenech et al. 2021; Vitali et al. 2024)—that were enough homogenous in terms of study population demographics—were subjected to quantitative evaluation (meta‐analysis) (Figure 1). Cohen's kappa value for global inter‐reviewer agreement was almost perfect, being 0.89. Characteristics and outcomes of included papers are reported in Table 2.

**Table 2:** Characteristics of the included studies.

N	Author	Year	Country	Tooth group	Patients’ number	Patient's age	Sex	N visit	Temporary dressing	Shaping Instrument	Coronal sealing
1	Farzaneh et al.	2018	Iran	First and second mandibular molars with irreversible pulpitis. No AP	110	28.56 ± 8.68	20M 35F	Single visit	NO	RaCe rotary instrument	ND
						28.34 ± 7.61	19M 36F				
2	Verma et al.	2019	India	First and second mandibular molars, non vital and AP	90	28.58 ± 7.61	21M 22F	Two visits	Calcium hydroxide paste + Cavit	Mtwo	Composite
						29.23 ± 7.93	18M 25F				
3	Mostafa et al.	2020	Egypt	First and second mandibular molars, non vital, with or without AP	303	32.14 ± 5.79	62M 92F	Two visits	No intracanal medication + Cavit	ProTaper Universal	Temporary filling
						31.60 ± 5.87	68M 86F				
4	Ulin et al.	2020	Sweden	All teeth, vital and non vital, with or without AP	211	51.6 ± 15.9	65M 87F	Single visit/two or more	Calcium hydroxide + zinc oxide‐eugenol cement	ProTaper, WaveOne, BioRace	Temporary filling (permanent restoration by referring dentist)
						51.5 ± 16.0	67M 78F				
5	Demenech et al.	2021	Brazil	All teeth, vital and non vital, with or without AP	169	34.7 ± 12.1	16M 27F	Single visit	NO	WaveOne Gold, ProDesign Logic	Glass ionomer cement
						37.8 ± 12.0	16M 26F				
6	Mokhtari et al.	2023	Iran	First and second mandibular molars with irreversible pulpitis. No AP	72	28.42 ± 9.27	8M 4F	Single visit	NO	Denco	Temporary filling
						31 ± 6.84	6M 6F				
7	Vitali et al.	2024	Brazil	First or second mandibular molars, non vital, with AP	154	33.51 ± 12.01	37M 40F	Single visit	NO	Reciproc	Composite
						33.75 ± 11.38	33M 44F				

Pre‐operative pain	Painkillers before RT	Painkillers after RT	NaOCl	Additional irrigation	Obturation material	Obturation technique	Pain scale	Follow‐up time	Main findings
NO	For at least 6 h before	Immediately after ibuprofen 400 mg. 10 pills more on demand	2.50%	EDTA 17%	Gutta and AH26	Cold lateral condensation	VAS	0–3–6–12 h 1–2–3–4–5–6–7 days	5.25% NaOCl was associated with significantly less pain than 2.5% of NaOCl, up to 3 days post‐treatment.
			5.25%						
YES	For at least 3 days before, antibiotics 1 month	ND	1%	EDTA 17%	Gutta and oxide‐eugenol based sealer	Cold lateral condensation	VAS	1–2–3–4–5–6–7 days	No statistically significant difference between 2 groups was observed at any time intervals.
			5%						
YES	At least 12 h	Analgesic on demand	1.3%	EDTA 15% gel and Carbamide peroxide	Gutta and Ah plus	Single‐cone	NRS	0–3 h 1–2–7 days from first visit	1.3% NaOCl was associated with less intense and less frequent post‐endodontic pain than 5.25% NaOCl.
			5.25%						
ES	ND	ND	0.5%	EDTA 15% Iodine‐potassium‐iodide 5%	Gutta and AH plus	ND	VAS	1–2–3–4–5–6–7 days. Results are reported as sum of pain at day 7	Frequency or magnitude of post‐operative pain was no significantly impacted by difference in the concentration of NaOCl (0.5% vs. 3%).
			3%						
YES	None	Analgesic on demand, every 12 h for 3 days	2.5%	EDTA 17%	Gutta and AH plus	Tagger's technique, thermomechanical	VAS	1–2–3 days	The 8.25% NaOCl solution was not significantly more associated with presence or intensity of pain than other solutions.
			5.25%						
YES	At least 12 h	NO	0.5%	EDTA 17%	Gutta and AH26	Cold lateral condensation	VAS	0–3 h 1–2–3–7 days	Pain intensity was not different in concentrations of 0.5% and 1%, respectively.
			1%						
NO	48 h before, antibiotics 1 month	Analgesic on demand	2.5%	EDTA 17%	Gutta and AH plus	Vertical condensation	NRS	3–6–12 h 1–2–3–7–14–30 days	The use of 8.25% NaOCl resulted in higher postoperative pain than the use of 2.5% NaOCl.
			8.25%						

### 3.2 Study Characteristics

All included papers were randomized clinical trials reporting on non‐vital permanent teeth or teeth with signs and symptoms of irreversible pulpitis, thus candidate to root canal therapy. Five studies enrolled first and second mandibular molars (Farzaneh et al. 2018; Verma et al. 2019; Mostafa et al. 2020; Mokhtari et al. 2023; Vitali et al. 2024), while 2 papers included all teeth (Ulin et al. 2020; Demenech et al. 2021). Clinical procedures and protocols were different among studies. Specifically, root canal treatment was performed in a single visit in the majority of the studies (Farzaneh et al. 2018; Demenech et al. 2021; Mokhtari et al. 2023; Vitali et al. 2024), whereas 2 studies have performed therapy in two visits (Verma et al. 2019; Mostafa et al. 2020), using only in 1 paper (Verma et al. 2019) calcium hydroxide paste as medicament. In the study conducted by Ulin et al. (2020), authors state that root treatment was obtained in one, two or even more visits, using calcium hydroxide as pulp dressing material. Root canal shaping was obtained with different systems of mechanical instrumentation and cold lateral condensation was the main used obturation technique (Farzaneh et al. 2018; Verma et al. 2019; Mokhtari et al. 2023), following by single cone (Mostafa et al. 2020), vertical condensation (Vitali et al. 2024) and hybrid technique (Demenech et al. 2021). Resin based sealer (AH Plus, Dentsply Sirona) was applied in 6 out 7 study whereas one study used zinc oxide based sealer (Verma et al. 2019). Regarding irrigation protocol, the concentration of NaOCl used in the included studies ranged from 0.5% to 8.25% (0.5%, 1%, 1.3%, 2.5%, 5%, 5.25%, 8.25%) and ethylenediaminetetra‐acetic acid (EDTA) (15% or 17%) was applied as additional irrigant in all studies. Each study compared different low and high NaOCl concentrations, that ranged from 0.5% to 2.5% and from 5% to 8.25%, respectively.

Disparities were noticed in all included articles regarding the type of coronal seal. Two studies have placed composite (Verma et al. 2019; Vitali et al. 2024), 1 study glass ionomer cement (Demenech et al. 2021) and other 3 papers reported used temporary restoration (Mostafa et al. 2020; Ulin et al. 2020; Mokhtari et al. 2023). Farzaneh et al. (2018). did not provide the type of coronal seal placed.

Regarding pain assessment, co‐founding factors as pre‐operative pain evaluation was reported in 5 out 7 studies (Verma et al. 2019; Mostafa et al. 2020; Ulin et al. 2020; Demenech et al. 2021; Mokhtari et al. 2023). Accordingly, pain killers were administrated from 6 h to 3 days before endodontic treatment. Pain was evaluated using numeric visual analogue scale (VAS) (Farzaneh et al. 2018; Verma et al. 2019; Ulin et al. 2020; Demenech et al. 2021; Mokhtari et al. 2023) and numerical rating scales (NRS) (Mostafa et al. 2020; Vitali et al. 2024) at different time points. The first pain evaluation ranged from 0 h (immediately post‐treatment) to 12 h. In 3 studies the first pain assessment was performed directly 24 h post‐therapy (Verma et al. 2019; Ulin et al. 2020; Demenech et al. 2021).

The maximum follow‐up time was 7 days for all studies (Farzaneh et al. 2018; Mostafa et al. 2020; Ulin et al. 2020; Mokhtari et al. 2023), except for Demenech et al (2021) that reported values at 3 days and Vitali et al. (2024) that additionally reported pain assessment at 14 and 30 days. The qualitative review suggested that four studies showed no significant difference in post‐operative pain using low and high concentration of NaOCl (Verma et al. 2019; Ulin et al. 2020; Demenech et al. 2021; Mokhtari et al. 2023). On the other hand, 2 studies reported less post‐operative pain when low concentration of NaOCl was adopted, respectively 1.3% versus 5.25% (Mostafa et al. 2020) and 2.5% versus 8.25% (Vitali et al. 2024). Finally, controversial results were showed by Farzaneh et al. (2018) reporting that 3 days after root canal therapy the use of 5.25% NaOCl was related to significantly less pain than 2.5%.

### 3.3 Risk of Bias

**Figure 2:**
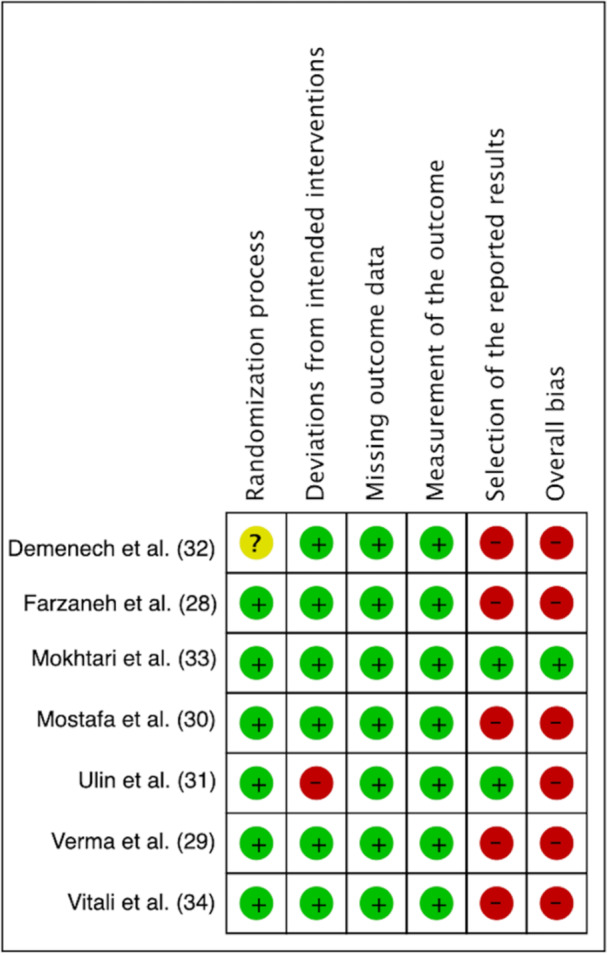
Review authors’ judgment about each risk of bias item for each included study.

**Figure 3:**
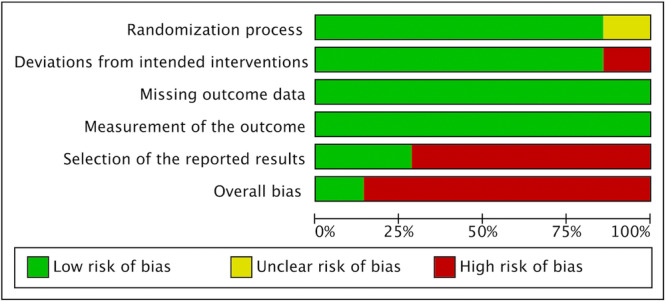
Review authors' judgment about each risk of bias item presented as percentages across all included studies.

The Cochrane Risk of Bias tool (RoB 2) was applied to assess quality of the included papers. Among them, 6 were categorized as having an overall high risk of bias (Farzaneh et al. 2018; Verma et al. 2019; Mostafa et al. 2020; Ulin et al. 2020; Demenech et al. 2021; Vitali et al. 2024) while the study conducted by Mokhtari et al. (2023) revealed an overall low risk (Figure 2). The shortcomings mostly concerned the deviations from the intended interventions (domain #2) for 1 study (Ulin et al. 2020) and the selection of the reported result (domain #5) in five papers (Farzaneh et al. 2018; Verma et al. 2019; Mostafa et al. 2020; Demenech et al. 2021; Vitali et al. 2024) (Figure 3).

### 3.4 Results of the Meta‐Analyses and Quality Assessment

**Figure 4:**
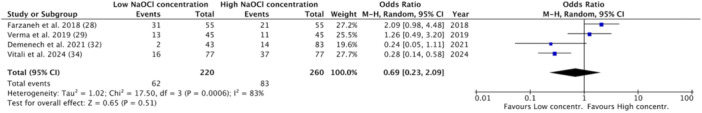
Forest plot of comparison: Pain after different NaOCl irrigation; outcome: Pain after 24 h.

**Figure 5:**
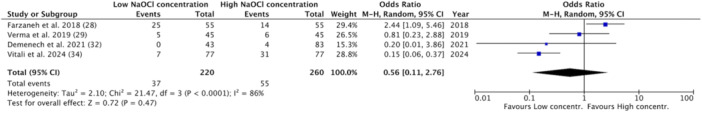
Forest plot of comparison: Pain after different NaOCl irrigation; outcome: Pain after 48 h.

**Figure 6:**
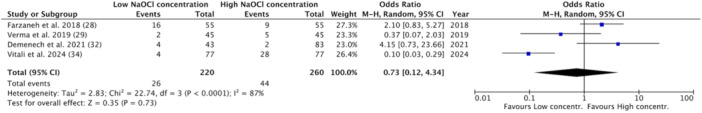
Forest plot of comparison: Pain after different NaOCl irrigation; outcome: Pain after 72 h.

Meta‐analyses were performed comparing the patients' perceived pain (presence or absence) when comparing different NaOCl concentrations in 3 different time‐frames: 24, 48, and 72 h after root canal treatment. The included studies were categorized based on low and high concentrations, using the following ranges as reference: low concentrations from 0.5% to 2.5% and high concentrations from 5% to 8.25%. The 3 meta‐analyses reported data of 480 patients for each time‐frame but none of them detected a statistically significant difference in perceived pain after using NaOCl at different concentrations (odds ratio: 0.69; 95% CI: 0.23–2.09; *p* value: 0.51, odds ratio: 0.56; 95% CI: 0.11–2.76; *p* value: 0.47, and odds ratio: 0.73; 95% CI: 0.12–4.34; *p* value: 0.73) at 24 (Figure 4), 48 (Figure 5) and 72 h (Figure 6), respectively. Conducted meta‐analyses reported notable heterogeneity according to *I* ^2^ values (*I* ^2^ = 83%, *I* ^2^ = 86%, and *I* ^2^ = 87%, respectively). The Grading of Recommendations, Assessment, Development, and Evaluations (GRADE) was used as a system to provide information on the certainty of the conclusions and the strength of the evidence (Table 3). Although the meta‐analyses drew conclusions from randomized clinical trials, considered to be the best‐available evidence, they were assessed to have only moderate strength of evidence because of high heterogeneity across all included studies and a general presence of wide confidence intervals in all meta‐analyses.

**Table 3:** GRADE on data included in the meta‐analyses.

Quality Assessment, Outcome: Pain after NaOCl irrigation at different concentrations						
Question: Among low and high concentration which one is more prone to cause pain after irrigation?						
Number of studies according to meta‐analysis (n)	Study design	Risk of bias	Inconsistency	Indirectness	Imprecision	Publication bias
n.4 Figure 4—after 24 h	RCT	High	Serious^a^	Not serious	Serious^b^	Undetected
n.4 Figure 5—after 48 h	RCT	High	Serious^a^	Not serious	Serious^b^	Undetected
n.4 Figure 6—after 72 h	RCT	High	Serious^a^	Not serious	Serious^b^	Undetected

^a^ Due to high heterogeneity.

^b^ Due to wide confidence intervals.

## 4 Discussion

Pain control is a critical aspect of root canal therapy and its subsequent stages (Venkata Teja et al. 2021). One major factor contributing to post‐operative pain is the improper usage of irrigants. NaOCl, known for its potent antimicrobial and tissue‐dissolving properties, is widely used in concentrations ranging from 0.5% to 8% (Teja et al. 2022). However, due to various reported complications, there is no consensus on the ideal concentration for clinical use. To the best of our knowledge, no systematic review and meta‐analysis have been published evaluating different concentrations of NaOCl and their effect on post‐operative pain until 72 h after treatment. Therefore, the current study aimed to evaluate the relationship between NaOCl concentration and pain occurrence following root canal treatment.

Meta‐analyses conducted at 24, 48, and 72 h after treatment revealed no significant differences in post‐operative pain between high and low NaOCl concentrations, although lower concentrations were linked to less pain occurrence. The use of NaOCl at higher concentrations enhances its antimicrobial activity and tissue‐dissolving capacity; however, it also increases cytotoxicity (Parirokh et al. 2012; Zehnder 2006). When extruded into the periapical tissues, NaOCl cytotoxicity may lead to patient discomfort, highlighting the importance of careful concentration selection and proper irrigation technique (Parirokh et al. 2012; Zehnder 2006; De Santis et al. 2022). Evaluation range of post‐treatment pain should at least consider 3 days, that's the interval time in which post‐endodontic flare‐up is mainly represented (Shamszadeh et al. 2020). Prasad et al. (2024) recently reported a significantly lower incidence of post‐endodontic pain when lower concentrations of NaOCl were applied at 24 and 48 h after treatment. Accordingly, the quantitative analyses in the present study extended pain assessment to 72 h.

Each of the included study compared different NaOCl concentrations categorized as “low” and “high” that ranged from 0.5% to 2.5% and from 5% to 8.25%, respectively. Although the considerable heterogeneity among studies—6/7 of the included papers reported an overall high risk of bias—a low concentration of NaOCl seemed to be associated with less pain, significantly in 2 papers (Mostafa et al. 2020; Vitali et al. 2024) and not significantly in 4 articles (Verma et al. 2019; Ulin et al. 2020; Demenech et al. 2021; Mokhtari et al. 2023). Only Farzaneh et al. (2018) observed significantly less pain using 5.25% NaOCl than of 2.5%, 3 days after root canal treatment. In addition, only 4 studies (Farzaneh et al. 2018; Verma et al. 2019; Demenech et al. 2021; Vitali et al. 2024) underwent quantitative evaluation, since 2 papers (Ulin et al. 2020; Mokhtari et al. 2023) provided comparison of different low concentrations of NaOCl (0.5% vs. 3% and 0.5% vs. 1%, respectively), and the study conducted by Mostafa et al. (2020) reported pain levels 1, 2 and 7 days after the first visit (i.e., irrigation and instrumentation) and on the second visit immediately after root filling, without any additional follow‐up time.

The inconsistencies between reported outcomes may be due to different variables that affect pain incidence, such as long preparation time, irrigation technique, instrumentation technique, used sealer and overfilling (Gupta et al. 2021). The same Authors stated that their outcomes might be attributed to the pre‐operative pulpal and periapical status of the included teeth. Indeed, Farzaneh et al. (2018). treated vital dental elements with inflamed pulps and no periapical reaction, therefore the presence of normal periapical structures seemed to avoid extrusion of the irrigants and debris as well as the use of 5.25% NaOCl had higher dissolution capacity of the remaining apical pulp that resulted in less inflammation. Additional co‐morbidities such as number of visits and intracanal medication might be related to inflammatory response (Emara et al. 2019; Erdem Hepsenoglu et al. 2018). Half of the papers included in the current systematic review reported root canal treatment in a single visit, while the other half in 2 appointments. Moreover, the intracanal dressing was not comparable and the effectiveness of coronal sealing was impossible to evaluate. These aspects might contribute to pain occurrence and should be deeply evaluated. The histopathological status of the pulp and the presence of peri‐apical tissue reaction also played an important role in the release of signaling molecules that might trigger the up‐regulation of inflammation and pain incidence (Guivarc'h et al. 2017; Xu et al. 2022; Iaculli et al. 2022).

Different pain assessment methods were used across studies, as NRS and VAS. Although the assessed pain levels were comparable, NRS generally showed higher compliance and responsiveness compared to VAS (Armogida et al. 2022). Future studies should ensure detailed reporting of pain assessment methods and consider the potential bias in self‐reported outcomes. It should be also considered variability in inclusion criteria among studies, related to pre‐operative pain presence as well as use of pain killers before treatment. There is a need of standardized criteria to reduce heterogeneity and future trials should maintain balanced baseline variables and minimize factors leading to post‐operative pain.

Although clinical effectiveness of NaOCl is evident, the proper concentration to obtain positive effects and to limit the drawbacks need to be further elucidated. Since the current evidence is limited, additional well‐designed trials with standardized methods are warranted to provide more reliable clinical guidance.

## 5 Conclusion

Within the limits of the current systematic review and meta‐analysis, it could be concluded that there was no difference in post‐endodontic pain levels using different concentrations of NaOCl. However, concentration of NaOCl < 5% might be associated with less pain incidence.

## Author Contributions

Study design and data extraction: Kavalipurapu Venkata Teja. Data extraction and interpretation: Kaligotla Apoorva Vasundhara. Meta‐analysis: Romeo Patini. Artworks and literature search: Salvatore Scolavino. Critical revision and results interpretation: Gianrico Spagnuolo. Search strategy and writing and editing of original manuscript: Flavia Iaculli. Search strategy, data interpretation and supervision: Mariangela Cernera.

## Funding

The authors received no specific funding for this work.

## Ethics Statement

The authors have nothing to report.

## Consent

The authors have nothing to report.

## Conflicts of Interest

The authors declare no conflicts of interest.

## Data Availability

Data are available upon request to the authors.
